# Supplementary material for: Modeling glioblastoma heterogeneity as a dynamic network of cell states
Source: Mol Syst Biol. 2021 Sep 16;17(9):e10105. doi: 10.15252/msb.202010105 (PMC8444284; doi:10.15252/msb.202010105)
Supplement: Supplementary file 6 — Source Data for Figure 5 [file MSB-17-e10105-s004.zip › Figure5A_sourcedata/GSEA_3017/nosigdb_stateD.GseaPreranked.1622795010397/NB_KI_CLUSTER7.html]

Details for gene set NB\_KI\_CLUSTER7[GSEA]

|  || Dataset | state23017 |
| Phenotype | NoPhenotypeAvailable |
| Upregulated in class | na\_pos |
| GeneSet | NB\_KI\_CLUSTER7 |
| Enrichment Score (ES) | 0.30874676 |
| Normalized Enrichment Score (NES) | 1.5769459 |
| Nominal p-value | 0.035928145 |
| FDR q-value | 0.17758887 |
| FWER p-Value | 0.328 |
Table: GSEA Results Summary

  

Fig 1: Enrichment plot: NB\_KI\_CLUSTER7      
 Profile of the Running ES Score & Positions of GeneSet Members on the Rank Ordered List

  

| PROBE | GENE SYMBOL | GENE\_TITLE | RANK IN GENE LIST | RANK METRIC SCORE | RUNNING ES | CORE ENRICHMENT || 1 | HMGCR |  |  | 141 | 0.262 | -0.0607 | Yes |
| 2 | AL359504.2 |  |  | 144 | 0.261 | -0.0149 | Yes |
| 3 | METTL4 |  |  | 195 | -0.253 | -0.0073 | Yes |
| 4 | GXYLT1 |  |  | 209 | -0.254 | 0.0288 | Yes |
| 5 | BTBD6 |  |  | 237 | -0.257 | 0.0548 | Yes |
| 6 | SASS6 |  |  | 261 | -0.260 | 0.0844 | Yes |
| 7 | HAUS5 |  |  | 303 | -0.264 | 0.1008 | Yes |
| 8 | PKP4 |  |  | 316 | -0.265 | 0.1397 | Yes |
| 9 | TMTC2 |  |  | 399 | -0.273 | 0.1263 | Yes |
| 10 | LMF2 |  |  | 425 | -0.276 | 0.1572 | Yes |
| 11 | SPPL2B |  |  | 432 | -0.277 | 0.2029 | Yes |
| 12 | TUBGCP5 |  |  | 443 | -0.279 | 0.2459 | Yes |
| 13 | TEDC1 |  |  | 462 | -0.281 | 0.2830 | Yes |
| 14 | NUP85 |  |  | 562 | -0.292 | 0.2601 | Yes |
| 15 | EED |  |  | 569 | -0.293 | 0.3087 | Yes |
| 16 | UBR7 |  |  | 767 | -0.336 | 0.2185 | No |
| 17 | CASK |  |  | 832 | -0.353 | 0.2334 | No |
| 18 | CCDC15 |  |  | 883 | -0.367 | 0.2616 | No |
| 19 | CEP295 |  |  | 974 | -0.404 | 0.2657 | No |
Table: GSEA details [plain text format]

  

Fig 2: NB\_KI\_CLUSTER7: Random ES distribution      
 Gene set null distribution of ES for **NB\_KI\_CLUSTER7**
